# What Does the Future Hold for Yellow Fever Virus? (I)

**DOI:** 10.3390/genes9060291

**Published:** 2018-06-08

**Authors:** Raphaëlle Klitting, Ernest A. Gould, Christophe Paupy, Xavier de Lamballerie

**Affiliations:** 1 Unité des Virus Émergents (UVE: Aix-Marseille Université, IRD 190, Inserm 1207, IHU Méditerranée Infection), 13385 Marseille Cedex 05, France; eag@ceh.ac.uk (E.A.G.); xavier.de-lamballerie@univ-amu.fr (X.d.L.); 2 UMR Maladies Infectieuses et Vecteurs: Écologie, Génétique Évolution et Contrôle (MIVEGEC: IRD, CNRS, Université Montpellier), 34394 Montpellier, France; christophe.paupy@ird.fr

**Keywords:** yellow fever virus, flavivirus, vector-borne transmission, emergence

## Abstract

The recent resurgence of yellow fever virus (YFV) activity in the tropical regions of Africa and South America has sparked renewed interest in this infamous arboviral disease. Yellow fever virus had been a human plague for centuries prior to the identification of its urban transmission vector, the *Aedes* (*Stegomyia*) *aegypti* (Linnaeus) mosquito species, and the development of an efficient live-attenuated vaccine, the YF-17D strain. The combination of vector-control measures and vaccination campaigns drastically reduced YFV incidence in humans on many occasions, but the virus never ceased to circulate in the forest, through its sylvatic invertebrate vector(s) and vertebrate host(s). Outbreaks recently reported in Central Africa (2015–2016) and Brazil (since late 2016), reached considerable proportions in terms of spatial distribution and total numbers of cases, with multiple exports, including to China. In turn, questions about the likeliness of occurrence of large urban YFV outbreaks in the Americas or of a successful import of YFV to Asia are currently resurfacing. This two-part review describes the current state of knowledge and gaps regarding the molecular biology and transmission dynamics of YFV, along with an overview of the tools that can be used to manage the disease at individual, local and global levels.

## 1. Introduction, a Historical Perspective

Yellow fever virus (YFV) is the type species of the genus *Flavivirus* (family *Flaviviridae*), owing its name to the jaundice associated with the liver dysfunction characteristic of clinically apparent human yellow fever. Although it is now commonly accepted that YFV originated in Africa [1] between 1500 and 3000 years ago [2,3,4] the first outbreaks of the “Black Death”, “Yellow Jack” or “Blood Vomit” (Xekik in Mayan) were reported in Barbados and in St. Christophe (now St. Kitts) in 1647 [5,6]. They were subsequently followed by the Yucatan epidemic (Cogolludo 1648) which was recorded more than a century before the first report of an African yellow fever (YF) epidemic in 1778 [7], that occurred among the British troops at St. Louis de Senegal [5,6].

Subsequently, until the end of the 19th century, outbreaks of YF disease were documented in port cities of North and South America, the Caribbean, Africa, and Europe, including the United Kingdom but particularly along the Mediterranean coast. Of note, YF outbreaks in Philadelphia (1793; 5000 deaths) [8] and, one century later, along the Mississippi River (1878, 120,000 cases including 20,000 deaths), were particularly large and deadly [9,10,11]. Outbreaks of YFV in North American and in European cities almost certainly resulted from multiple introductions of the virus off the ships that traded with Africa, the Caribbean, and South America. In other words, this virus was never endemic but “pseudo-epidemic” in these cities. [5,12,13,14,15,16]. Presumably, this was also true in South America with the first introductions of YFV but the indigenous mosquitoes and other wildlife provided suitable hosts for the virus to spill back into an enzootic cycle allowing viral maintenance in the South American forests and surrounding savannah regions.

Until the end of the 19th century, the etiology of yellow fever was not clearly defined and frequently referred to as a “miasma” transmitted by foul air [11]. In the 1890s, the British YF commission (W. Myers and H.E. Durham) and the commission established by the United States (US) Government, led by W. Reed, were successively sent to Cuba to investigate the mode of YF disease transmission, that caused high mortality rates among soldiers at that time [17]. In coherence with previous hypotheses [11,18,19,20,21,22,23,24,25], the Reed Commission established that the inter-human transmission of YF was supported by mosquitoes and particularly by *Aedes* (*Stegomyia*) *aegypti* (Linnaeus) [26,27]. In a series of human infection studies involving both members of the Commission and military “volunteers”, the mosquito-borne transmission of YF was established. It was formally observed that mosquitoes transmitted the disease from infected patients to healthy study participants, sometimes at the cost of their lives [28]. Such sacrifices were not made in vain as, in less than a year, YF was successfully controlled in Cuba thanks to the drastic measures implemented by William Gorgas which involved vector control (elimination of both larvae and adult mosquitoes), quarantine of active YFV cases, and the surveillance of port/city entrance [5]. The origin of this discovery remains controversial, as Reed’s achievement may have greatly benefited from the work of J. W. Lazear, a member of Reed’s commission who died in September 1900. It has recently been argued that he was the first person to demonstrate transmission of the infectious agent of YF from an infected to a non-immune individual through the bite of a mosquito (*Culex fasciatus* sp. now recognized as *Ae. aegypti*) [29]. Either way, the landmark experiments of the Reed Commission produced the first evidence that viruses could be transmitted by arthropods, thus opening the fields of arbovirology and medical entomology. In 1898, the concept of “virus” did not exist, and, as bacteria were occasionally identified in cultures from YF patients, the etiological agent of YF disease was thought to be a bacterium (*Bacillus icteroides* or *Leptospira icteroides*) [30,31]. Two years later, the agent responsible for YF disease was shown to be filterable [28].

In 1928, Stokes and colleagues reported the identification of the transmissible component of YF. The infectious YFV was isolated following the inoculation of a rhesus macaque with the blood of a patient named Mr. Asibi, in 1927 in Ghana. This recovered viral agent was efficiently used for both human-to-monkey and monkey-to-monkey transmission with injections of blood or serum. In addition, infection experiments confirmed *Ae. aegypti* efficiently transmitted the virus from viraemic to naive monkeys [32,33]. The French YF strain was also isolated in 1927, in Dakar (Pasteur Institute, Senegal, West Africa), from a Syrian patient named Mayali, who presented with mild YF [34].

A few years later, in 1930, the first laboratory animal experiments in virology showed that newborn mice died following intracerebral inoculation with YFV, after viral dissemination to the brain, spinal cord, peripheral nerves and adrenal glands [35]. Theiler also described how the virus could be indefinitely propagated in mice by intracerebral injection of infected mouse brain. Furthermore, he observed that repeated passages of YFV through mouse brains led to a gradual loss of virulence for rhesus monkeys [35]. The first YFV in vitro culture was achieved two years later, using Carrell dishes [36] and several kinds of tissue notably from kidneys or testicles of guinea pigs, and rabbits as well as chicken embryos [37]. Although Theiler had already made substantial contributions to the field of YFV research, his greatest achievement was the development of the vaccine strain YF 17D, through in vitro serial passages of the virulent strain *Asibi* (see above) in chicken embryo tissues [38,39]. In 1951, the development of YF vaccine earned Theiler the first and so far the only Nobel Prize given for the development of a viral vaccine. This live-attenuated vaccine was used as early as 1937 in Brazil and two of its substrains, 17DD and 17D-204, at pre-determined passage numbers, are still used for the manufacture of the vaccine. The substrain YFV 17DD exclusively serves for vaccine production in Brazil while in the USA and the Old World, the substrain 17D-204 is used. Both 17D-204 and 17DD-seeded vaccines are widely employed for immunization against YFV. The yellow fever vaccine remains one of the safest and most efficacious vaccines ever produced [40,41].

YFV currently circulates in the tropical regions of Africa and South America where it is primarily maintained through a sylvatic cycle between its non-human primate hosts and sylvatic mosquito vectors. Yellow fever virus transmission relies on distinct hosts and vectors in the Old and the New World and these differently shape both the evolution and the dissemination of the virus. In the Old World (Africa), YFV transmission can be sylvatic, rural, or urban, with occasional large outbreaks that arise in urbanized regions, as recently observed in Angola and the Democratic Republic of Congo in 2015–2016. By contrast, in the New World, YFV mainly spreads through sylvatic transmission cycles involving non-human primates, with occasional human cases, mostly found in rural or peri-urban localities, as illustrated by the last outbreak that started in Brazil, in December 2016. There are still grey areas in our understanding of the mechanisms that underlie YFV maintenance and dissemination. These are tightly linked to crucial questions about the likeliness of occurrence of large urban YFV outbreaks in the Americas and of a successful import of YFV to Asia. The first part of this review on YFV outlines the main aspects of YFV maintenance and transmission and describes the state of our knowledge regarding YFV ecology, phylogeny, and recent epidemiology.

## 2. Ecology of Yellow Fever Virus

**Figure 1:**
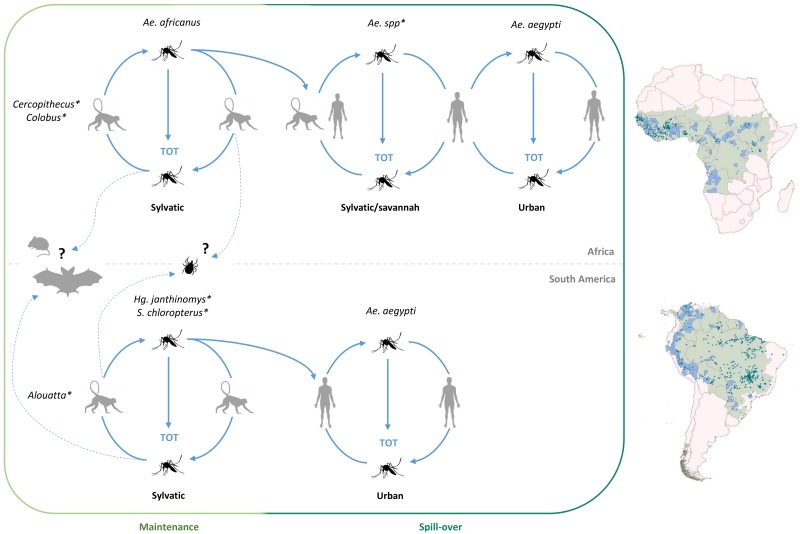
Ecology of Yellow Fever Virus (YFV) (modified from [51,52]). Areas with autochthonous vector-borne transmission are highlighted in pale green. A star indicates that the contributing vectors are detailed in the caption. Yellow fever virus maintenance in nature is thought to be ensured through a sylvatic cycle between its non-human primate (NHP) hosts and its sylvatic vectors in Africa and South America. Comprehensive lists of the African/South American arthropods for which YFV isolation and/or experimental transmission has been reported are provided in the Supplementary Table S1. In Africa, NHPs belong to the genera *Cercopithecus*, *Colobus*, and *Galago* [44,45,46]. In Africa, the main sylvatic vector is *Aedes* (*Stegomyia*) *africanus* (Theobald) while at the fringe of forested areas several other *Aedes* species may contribute to the intermediate sylvatic/savannah cycles, which involve both human and non-human primates. These may also occasionally participate in the sylvatic cycle. They notably include *Aedes* (*Stegomyia*) *bromeliae* (Theobald) (belonging to the *Aedes* (*Stegomyia*) *simpsoni* complex), *Aedes* (*Stegomyia*) *opok* Corbet and van Someren, *Aedes* (*Diceromyia*) *furcifer* (Edwards) and *Aedes* (*Diceromyia*) *taylori* Edwards, *Aedes* (*Fredwarsius*) *vittatus* (Bigot), *Aedes* (*Stegomyia*) *luteocephalus* (Newstead) and possibly *Aedes* (*Stegomyia*) *aegypti* (Linnaeus). Yellow fever virus can finally spread to urban areas and start large urban and periurban epidemics vectored by the domestic vector, *Ae. aegypti*. In South America, YFV has been identified in NHPs from the genera *Alouatta* (main host), *Saimiri*, *Ateles*, *Aotus*, *Cebus*, *Callicebus*, *Callithrix*, and *Saguinus* [53,54,55,56,57]. The sylvatic vectors include species from the genera *Haemagogus* and *Sabethes* notably *Haemagogus* (*Haemagogus*) *janthinomys* Dyar, *Haemogogus* (*Conopostegus*) *leucocelanus* (Dyar and Shannon), *Haemagogus* (*Haemagogus*) *Spegazzinii* Brethes, *Sabethes* (*Sabethoides*) *Chloropterus* (Von Humboldt), *Sabethes* (*Sabethes*) *Albipivus* Theobald and *Sabethes* (*Sabethes*) *Cyaneus* (Fabricius). To date, the only domestic vector that has been clearly identified in Southern America for YFV is *Ae. aegypti*. Transovarial transmission (TOT) of YFV in mosquitoes has been reported and also participates in YFV natural upkeep, although its epidemiological importance is still debated [51,58,59]. Additional compatible hosts (bats, rodents) and vectors (ticks) have been identified and may take part in alternative transmission/maintenance cycles [45,60,61,62]. Africa and South America maps showing YFV occurrence and risk zones have been reused from Shearer and colleagues [52] (CC BY 4.0). Green dots correspond to case reports from locations smaller than 5 × 5 km in area, a blue shade to case reports from locations over 5 × 5 km in area and a pale green shade, to contemporary risk zones as defined by Jentes and colleagues [63].

Yellow fever virus is endemic in the tropical regions of sub-Saharan Africa and South-America, where its natural circulation is conditioned by the presence of both its mosquito vectors and primate hosts. In Africa, YFV is maintained in enzootic cycles involving sylvatic vectors (mainly from the *Aedes* (*Stegomyia*) *africanus* group species but secondarily from other *Aedes* species belonging to several subgenera) and non-human primates (NHP) notably from *Cercopithecus* and *Colobus* genera (sylvatic cycle) [42,43,44,45,46]. Emergence of the virus occurs if humans become infected when bitten by sylvatic mosquitoes that previously fed on viraemic monkeys notably during the occasional circulation of sylvatic mosquito populations into villages found at the fringe of forested areas. In rural areas, also referred to as the “Zone of emergence”, small-scale outbreaks (sylvatic/savannah cycles) can locally involve peridomestic “bridge” mosquito vectors from the genus *Aedes* [47,48,49]. Such intermediates cycles may settle when (i) humans get infected in the forest through the bite of a sylvatic mosquito and bring back the virus into their village where secondary transmission is ensured by peridomestic mosquito populations; (ii) the virus is introduced into plantations by viraemic monkeys on which peridomestic mosquito populations may feed. In West Africa, the further spread of YFV to dry, more populated areas with high densities of the highly anthropophilic *Ae. aegypti* mosquitoes can lead to larger outbreaks in periurban and urban areas (urban cycles) [50]. As an additional mean for YF maintenance, transovarial transmission (TOT) in the mosquito vector may also contribute to the spread of the virus. All these cycles are detailed in Figure 1.

### 2.1. In Africa

YFV most likely evolved in the rainforests of Central Africa (see Section 3) where it is still maintained in a primary cycle between NHPs and sylvatic mosquitoes that breed in tree holes, including *Aedes* (*Stegomyia*) *africanus* (Theobald), and related species such as *Aedes* (*Stegomyia*) *opok* Corbet and van Someren. Cycles involving *Ae. africanus* species were first described in Uganda in 1948 and the virus has since been shown to circulate in forested areas throughout Africa [47,64]. *Aedes opok* is found in higher densities than *Ae. africanus* in the forested regions of central Africa [65] but both species participate in the local spread of the virus, displaying similar transmission dynamics with limited sporadic human cases [65,66,67]. Importantly, the rural/periurban, anthropophilic, *Aedes* (*Stegomyia*) *albopictus* (Skuse) or “the Asian tiger mosquito” is now present in several countries of Central Africa [68,69], where it has been reported to enable the transmission of chikungunya and dengue viruses [70,71,72]. As American and European *Ae. albopictus* populations have shown to be experimentally competent for YFV [73,74,75], a future possible participation of this vector in YFV transmission in Central Africa should be monitored carefully. Of note, a large urban epidemic (>900 confirmed cases) was reported in December 2015 in Angola, from where it spread rapidly to the Democratic Republic of Congo, circulating until October 2016 [63,64]. While the primary vector of the epidemic has not been formally identified, it seems likely that bridge vectors (i.e., peridomestic) such as *Aedes* (*Stegomyia*) *simpsoni* (Theobald), have introduced YFV to the urban/peri-urban areas and the majority of cases in, or close to the urban areas, are likely to have been vectored by domestic *Ae. aegypti*. A joint analysis of datasets describing vector suitability, human demography, mobility in central Africa and the epidemic itself identified spatial associations between the risk of YFV invasion and local environmental suitability for the *Ae. aegypti* mosquito [64].

In East Africa, YFV is maintained in a sylvatic cycle similar to that described in Central Africa, from which it may periodically emerge in intermediate sylvatic/savannah cycles [76,77]. The cycles involve both NHP-mosquito-human and human-mosquito-human transmissions and most often, cause limited outbreaks. The emergence of YFV from the sylvatic environment occurs in the so-called “Zone of Emergence” (moist savannah and forest/savannah ecotones) [42], and such events are vectored by a variety of *Aedes* species including *Aedes* (*Stegomyia*) *bromeliae* (Theobald) [78]. This mosquito was identified as an important bridge vector in banana plantations close to forested areas in Uganda where it was observed to feed on both humans and NHPs [49]. The large YF outbreak of 1960, in Ethiopia, constitutes a good example of the epidemic transmission capacity of this vector [79]. Several other vector species are associated with YF maintenance and transmission, as suggested by direct isolation of virus from multiple species, population dynamics, geographic distribution, behavior and/or the ability of the mosquito to transmit YFV under laboratory conditions [80,81,82,83,84]. Although East and Central Africa account for the greatest taxonomic diversity of vectors associated with YF, it is problematic to determine whether or not a given mosquito species is a YF-competent vector in nature [85]. As an example, the 2013 Ethiopia outbreak may be one of the first to be reported as involving *Ae. aegypti* as a vector in East Africa [86].

As in Central and East Africa, *Ae. africanus* remains the main sylvatic vector for YFV in West Africa. In contrast, different YF-competent *Aedes* mosquitoes flourish in the areas surrounding the forest and the savannah of West Africa, including *Aedes* (*Diceromyia*) *furcifer* (Edwards) and *Aedes* (*Diceromyia*) *taylori* Edwards, *Aedes* (*Stegomyia*) *luteocephalus* (Newstead), *Aedes* (*Fredwarsius*) *vittatus* (Bigot), and *Aedes* (*Stegomyia*) *metallicus* (Edwards) species [58,87]. These species reach particularly high densities during the rainy and early dry seasons and are associated with transmission among NHPs, notably in large gallery forests. They also play a major role in transmission, from NHPs to humans and among humans, who may then become the dominant host in sylvatic/savannah cycles (see Figure 1). During the dry seasons, the transmission may then shift to the domestic *Ae. aegypti* vector, as illustrated by the 1978–1979 Gambia epidemic [50]. The spread of the virus from the forest-savannah ecotone to moist/dry savannah is ensured by viraemic humans moving from one environment to another, with dissemination being constrained by human population densities and also bridge and/or urban vector densities. Despite their apparently poor vector competence under experimental laboratory conditions [88], *Ae. aegypti* mosquitoes are notably anthropophilic, with a unique capacity to reach high densities in urban areas, therefore with a significant vector capacity. Indeed, *Ae. aegypti* has “domesticated” and adapted to breeding in dry urban areas where domestic water containers, scrap tires, discarded cans, waste plastic vessels, etc., provide a plethora of sites for their proliferation and where they have established dominance over all other anthropophilic *Aedes* species vectors. Accordingly, they are associated with explosive *Ae. aegypti*-borne epidemics with high prevalence, of which the first detailed examples in Africa are those that occurred in 1962–1965 and in 1969–1970 in Ethiopia, Senegal, and Nigeria [48,89].

Transovarial transmission (TOT) of YFV via its mosquito vectors [51] has also been suggested as an additional mode of YFV maintenance. Evidence from field studies and laboratory TOT experiments [90,91,92,93,94,95,96] support the hypothesis that TOT could have a relative role in YFV maintenance in East and Central Africa, notably for its long-term survival in seasonally drier habitats. It has been proposed that vertical transmission may also account for the slow evolution of YFV [1,51,97,98]. Tick-borne flaviviruses also have relatively low evolutionary rates, which are thought to result, at least partly, from the long periods between the feeding stages during which the virus appears to be relatively quiescent in the resting/diapausing tick [97,99]. However, when the tick feeds, virus infectivity increases by orders of magnitude in the salivary gland presumably increasing the likelihood of virus survival during the transstadial phase, prior to the next period of quiescence [100]. By analogy, YFV enters a quiescent state during egg-survival which may be extended to months or years prior to hatching, maturation, and subsequent replication and transmission as the infected newly emerging mosquitoes take their first meal. However, several elements, notably the low rates of infection of the progeny reported under laboratory conditions (maximum 1:500), indicate that TOT alone is not likely to account for long-term YFV maintenance [58,101]. The deleterious impact of YFV infection on mosquito survival and development additionally rules out TOT as a unique mechanism for YFV maintenance in nature [59,92]. Rather, YFV may be maintained primarily through amplification in primates and transmission to new susceptible hosts. The TOT mode of survival and transmission would apply particularly under adverse conditions and probably only for a limited number of mosquito generations [58].

### 2.2. In South America

As will be discussed further in this review, Africa is now recognized as the evolutionary cradle of YFV. Hence, it is almost certain that YFV did not occur first in a sylvatic cycle in the Americas. However, after its introduction into the New World, the virus efficiently spilled back into a sylvatic cycle that involved species of hosts and vectors that were completely new to the virus [85].

YFV circulation has been documented in numerous regions of the Caribbean and South America (e.g., Cuba, Trinidad, Brazil, Argentina, Paraguay, Venezuela, Bolivia, Colombia, and Peru) and was notably described in spider monkeys (*Ateles* sp.), owl monkeys (*Aotus* sp.), squirrel monkeys (*Saïmiri* genus) and howler monkeys from the genus *Alouatta* [53,54,55,56,57,58,102]. Importantly, in these species, lethality was reported, reaching degrees of severity that were never observed in monkeys from the African continent [103,104]. Such susceptibility to severe YFV infection may reflect the relatively recent introduction of the virus into the Americas [2]. In this case, the virus would be likely to evolve towards a decreased pathogenicity in these species. In the meantime, as they often precede the occurrence of human cases, epizootics among NHPs are known to serve as a surveillance or early warning system [2,51,104].

In South America, non-urban YFV circulation involves mosquito species in the genera *Haemagogus* and *Sabethes* that ensure transmission among NHPs and “spillover” from NHPs to humans. A large variety of species is involved in the sylvatic transmission cycle, notably *Haemagogus* (*Haemagogus*) *janthinomys*, *Haemogogus* (*Conopostegus*) *leucocelanus* (Dyar and Shannon), and *Sabethes* (*Sabethoides*) *Chloropterus* (Von Humboldt) [49,85,105,106,107,108,109]. *Aedes aegypti* was identified as the primary urban vector for YFV while *Haemagogus* is not considered to be a significant vector of yellow fever in urban settings. It is not clear whether the new mosquito vectors of YFV (e.g., *Haemagogus*, and *Sabethes*) were naturally competent or if the virus adapted progressively to these species. A gradual “back to the forest”, “spill-back” dispersal of the virus up the chain of transmission would be the most likely scenario. Historically, in the New World, *Ae. aegypti* was the first YFV vector species to be identified [28]. At that time, large urban YF epidemics, vectored by *Ae. aegypti* mosquitoes occurred in port cities of South and Central America, the Caribbean, and the United States. These occurred upon the arrival of slave-trading ships from West Africa, from where the virus was inadvertently being exported together with its domestic vector *Ae. aegypti*. In the tropical/sub-tropical regions of South America, YFV was thus introduced to large non-immune populations among which the virus would be dispersed, gradually spreading to the nearby countryside and forest environment. These infected humans could seed rural and sylvatic mosquitoes that may feed on non-human primates, thus initiating a sylvatic transmission cycle. In contrast, in the non-tropical regions of North America, explosive epidemics in urban areas during warm summers would rapidly decline as temperatures dropped below the critical level for mosquito transmission and survival. The risk of further epidemics was therefore entirely dependent on the arrival of YFV and *Ae aegypti-*infested ships during subsequent summers. The well-implemented vertical “top-down” vector-control campaigns that occurred in the tropical and sub-tropical Americas between the 1900s and 1940s [110] and from the mid-1940s to the 1970s [111] effected major but temporary reduction of *Ae. aegypti* mosquito populations and the eradication of urban YFV in numerous countries of the Caribbean, Central and South America (Pan-American Health Organization, PAHO, 1967) such as Cuba [112]. However, *Ae. aegypti* and its related diseases (as dengue) re-emerged in the Americas and it soon became apparent that in countries where YFV had established sylvatic cycles, rural outbreaks with the potential to initiate urban epidemics could not be totally controlled [110]. The control measures may also have restricted YFV to sylvatic cycles favoring the expansion of YFV lineages that were well-adapted to sylvatic *Haemagogus* and *Sabethes* species rather than to *Ae. aegypti*.

South American NHPs are highly susceptible to infection by YFV, leading to significant morbidity and mortality. Thus, viral amplification and dissemination among NHPs is pronounced. In contrast in the forest environment, there is a low incidence of monkey-mosquito-human and human-mosquito-human transmission, with an annual incidence of reported YF cases throughout South America, that rarely exceeds 500 cases/year [113]. In Latin America, the mechanism of YFV maintenance is not completely understood. On the one hand, sylvatic transmission of the virus depends on the renewal of susceptible non-human primate populations such as *Alouatta* monkeys, but the latter can suffer large losses following epizootic outbreaks. Such a mechanism accords with the periodic pattern of YFV outbreaks that has been reported, notably in Brazil (e.g., Goiás state), with five to seven year time lapses that correspond to the renewal of susceptible NHP populations necessary for viral amplification [114]. On the other hand, as in Africa, even when considering the possibility of vertical transmission, viral amplification by the vector population alone is rather unlikely. Hence, YFV would need to be present in several species of NHPs with diverse susceptibility and mobility patterns [104]. Thus, it would persist and circulate in specific sylvatic regions of South America, depending on the movements of NHP populations [2]. Indirect support for this scenario can be found in a recent study which showed a strong association between primate diversity and the presence of YF human cases [115]. In some instances, molecular data have produced evidence of viral spread over 2000 km (viz. between the states of Pará and Goiás/Bahia), implying a mechanism for transportation different from NHP population migration [116]. In such situations, movement of pauci-symptomatic, infected humans, or illegal traffic of infected wild animals, could be incriminated [114].

### 2.3. Heterogeneous Populations within the Domestic Vector Species *Aedes (Stegomyia) Aegypti* (Linnaeus)

**Figure 2:**
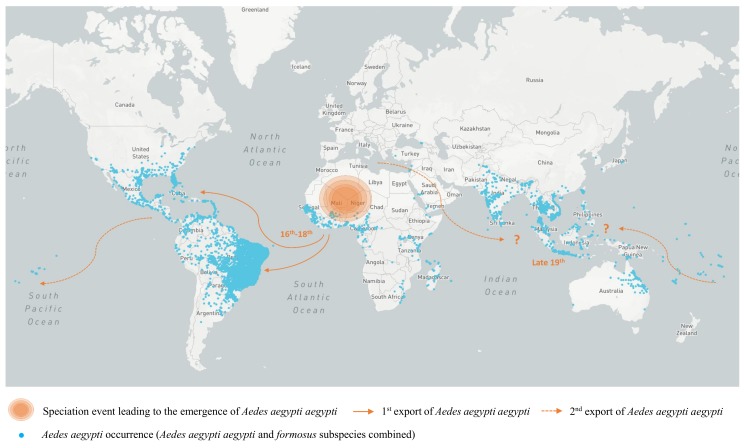
Global dissemination of *Ae. aegypti* species. *Ae. aegypti* (Aaa and Aaf subspecies combined) occurrences (adults, pupae, larvae or eggs) are indicated by blue dots. Occurrence data have been retrieved from the global geographic database of known occurrences of *Ae. aegypti* between 1960 and 2014 compiled by Kraemer and colleagues [137]. The Aaa subspecies most likely emerged during a single sub-speciation event around 4000 years ago, during the severe drying events that accompanied the expansion of the Sahara in the Northern part of Africa [4,119,120,121]. As confirmed through several genetic analyses performed using either nuclear or mitochondrial markers and microsatellite loci [119,120,121], Aaa was exported to the Americas during the slave trade. By the end of the 19th century, it was probably introduced from America into Asia. Possible additional introductions from the Mediterranean region may also have contributed to the colonization of Asia by this mosquito [4,120,124,125].

As for all arboviruses, YFV dissemination is tightly linked to the presence of competent mosquito vectors, the most recognized of which is the “pure” or “light” colored form of the domestic mosquito vector of YFV, *Aedes aegypti aegypti* (Aaa) (see Figure 2) [4]. Its ancestor originated in Africa, bred in tree holes and fed on non-human animals, with ecological patterns similar to those of the contemporary “black” colored form, *Aedes aegypti formosus* mosquito (Aaf) [4,117,118]. The allopatric speciation between the Aaa and the Aaf forms of *Ae. aegypti* most likely occurred as a single sub-speciation event around 4000 years ago, during the severe drying events that accompanied the expansion of the Sahara in the Northern part of Africa [4,119,120,121]. Multiple domestication events probably allowed the subsequent selection for “domestic” tendencies such as the ability to exploit artificial water storage elements created by humans or that of feeding on humans [122]. It was first suggested that Aaa had been repeatedly exported to the Americas during the slave trade [4]. This was then confirmed through several genetic analyses performed using either nuclear or mitochondrial markers and microsatellite loci [119,120,121,123]. Genetic analyses also indicate that the New World may have been the source for the introduction of Aaa into Asia, where the subspecies landed by the end of the 19th century and subsequently dispersed throughout urban areas (see Figure 2). Additional introductions of Aaa from the Mediterranean region may also have contributed to the colonization of Asia by this subspecies [4,120,124,125]. While both the virus and its vector were successfully introduced to the Americas during the slave trade, this was not the case upon the importation of Aaa into Asia.

At the present time, *Ae. aegypti* populations are found in Asia, America, Africa, Oceania, the Eastern Mediterranean and the Red Sea coasts. In Africa, populations mainly correspond to the Aaf subspecies [120,124,125,126] and the presence of Aaa was only reported in some coastal areas in West and East Africa (Senegal and Kenya) [118,120]. This could result from a secondary introduction from the Americas through shipping or from the preservation of a replicate form from the parent, Aaa [117,118]. 

In Latin America, *Ae. aegypti aegypti* was virtually eradicated during the 1940s–1970s but completely reinfested the region from both the south of the continent and the Caribbean, where residual mosquito populations subsisted [120,126,127,128]. During the ‘eradication’ period of Aaa in South America, YFV could only circulate in sylvatic mosquito species, mostly from the *Haemagogus* and *Sabethes* genera (see Section 2.2). The extended circulation of YFV in these mosquito populations may have resulted in the selection of lineages adapted to sylvan mosquitoes which may in-turn, replicate and spread less efficiently in Aaa [129]. Although appraising vector-competence is challenging, the apparent susceptibility to YFV infection of Aaa mosquitoes and their ability to transmit the virus under laboratory conditions [75] suggest that a differential adaptation alone may not account for the absence of Aaa-borne outbreaks since the middle of the 20th century in the region [85,126]. Other factors, such as mosquito feeding behavior and habitat preferences, are also key for vector capacity and involvement in *spillover* and/or inter-human disease transmission. Finally, out-competition by dengue virus has also been proposed as a part of the answer, as YFV is a highly virulent and primarily sylvatic virus, which is less prone to epidemic spread than dengue virus [51,130]. This phenomenon could take place into the host (cross-protection) and/or the vector (outcompetition). However, both field and experimental data remain too sparse and sometimes diverge [131,132] and more investigations are definitely needed so that these assumptions can be discussed on a solid basis. Overall, the risk of urban spread of YFV due to the presence of dense populations of Aaa mosquitoes in numerous urban centers where most of the inhabitants are nonimmune remains a concern in multiple countries in South America [75,133,134,135,136].

## 3. Evolution and Dispersal of Yellow Fever Virus Strains

### 3.1. Phylogeny

**Figure 3:**
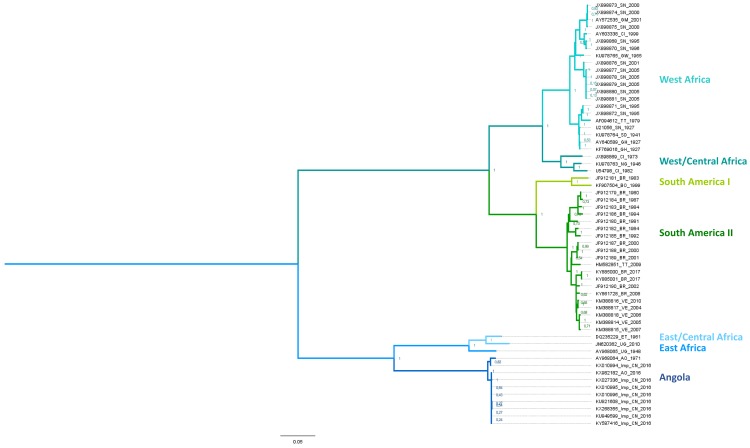
Phylogenetic relationships among strains of YFV. The tree was inferred from an alignment of 59 YFV coding sequence (CDS) downloaded from the European Molecular Biology Laboratory (EMBL) database and aligned according to amino acid sequences using clustalW as implemented in MEGA 7.0 software, v.7.0.26. Phylogenetic reconstruction was done using a maximum-likelihood (ML) method (General Time Reversible Model with a discrete gamma distribution of rates across sites (5 categories (+G, parameter = 0.8110)) and invariant sites ([+I], 24.06% sites)) and bootstrap resampling with 1000 replicates on MEGA 7.0 software. The tree with the highest log likelihood (−69934.65) is shown. Initial tree(s) for the heuristic search were obtained automatically by applying Neighbor-Join and BioNJ algorithms to a matrix of pairwise distances estimated using the Maximum Composite Likelihood (MCL) approach, and then selecting the topology with superior log likelihood value. The tree is drawn to scale, with branch lengths measured in the number of substitutions per site. The percentage of trees in which the associated taxa clustered together is shown next to the branches. Sepik virus (Genbank accession number: NC008719) was used as an outgroup.

Only one serotype has been identified within the Yellow Fever virus species. However, seven genotypes (see Figure 3), have been defined as “distinct lineages which differ by greater than 9% at the nucleotide sequence level” [138]. The five African lineages include two West African genotypes (West/Central and West; former I and II), a single Central/South African genotype (Angola) and two East African genotypes (East and East/Central) [138]. In addition, there are two South American genotypes (I and II) which were derived from an ancestral West African lineage. Phylogenetic studies on both the partial and complete sequences of YFV strains have provided valuable data for understanding the epidemiology of YFV in Africa and South America [51,138,139,140,141,142,143].

The five African YFV genotypes are characterized by their specific areas of circulation. The West Africa genotype I was recently redefined as the West/Central genotype and groups together with strains that circulate in Nigeria, Cameroun, and Gabon. The new Western genotype, former West Africa II, includes strains from Senegal, Guinea, Ghana, and the Ivory Coast (see Figure 3). The East Africa genotype corresponds to strains circulating in Uganda and Sudan, and the East/Central Africa genotype includes strains from the Central African Republic, Democratic Republic of Congo, and Ethiopia. Finally, the Central/South African (or Angolan) genotype mostly comprises strains from Angola [144]. The most recent common ancestor of all genotypes is estimated to have emerged between 700 and 1200 years before the first genotypes emerged [1,98,144], apparently by dispersal from Central Africa. The West and East African genotypes are the most recently emerged lineages and the most geographically distant from central Africa. This evolutionary pattern shows dispersal of the virus away from its evolutionary cradle. The existing molecular data regarding genotype circulation in Africa show dynamics that are constrained by the presence of both hosts and vectors, with the formation of discrete epidemic foci [143,144]. However, some examples of co-circulation can be found in the outbreaks of 1983 and 1985 in Burkina Faso, caused by the Western and West/Central genotypes, respectively [143].

As indicated above, the South American genotypes derive from West Africa and most probably diverged during the slave trade [1]. They had probably emerged by the end of the 19th century [145] and subsequently diversified to produce several lineages that are associated with distinct geographic areas (Brazil, Colombia, Bolivia, Peru, Trinidad/Tobago and Venezuela). Yellow fever virus circulation dynamics in South America (and the Caribbean) are characterized by both local circulation associated with in situ evolution [139,146] and occasional circulation across Latin American countries [56,139] with frequent lineage replacement from outbreak to outbreak. Lineage replacement is a particularly important phenomenon in the evolution of South American genotype I [116]. In this genotype, the “Old” lineage, comprises the Old Para, 1A, 1B, and 1C sub-lineages that have progressively been replaced by “Modern” sublineages. The currently prevailing “Modern” lineage emerged in Trinidad and Tobago in the 1980s and now consists of the Trinidad and Tobago, 1D and 1E sub-lineages [146,147]. Similarly, lineage replacement occurred within the “Modern” lineage as observed during the epidemic of 2008–2009 in Rio Grande do Sul and Sao Paulo. This outbreak was caused by the emergent sub-lineage 1E, that successfully replaced its ancestral sub-lineage, 1D and now prevails in the region [109,145,146].

Much remains to be learned concerning the circulation dynamics of YFV in Africa and the Americas largely because the data available for phylogenetic analysis of YFV isolates are scarce and viral samples are rarely preserved for genetic studies [146,148,149].

### 3.2. Emergence Out of Africa

The separation of the American and the African continent occurred during the break up of Pangea, 150 million years ago, long before the probable time of emergence of YFV species. This implies that YFV must have appeared in one of the two continents before spreading to the other [149]. Several lines of evidence indicate that the most probable evolutionary scheme for YFV emergence is that of an African origin, which is probably common to all mosquito-borne flaviviruses, as proposed in 2001 [150]. Early analyses of YFV nucleotide sequences suggested that the virus emerged at least thousands of years ago [1,151], in Africa.

Phylogenetic studies of rates of nucleotide substitution and divergence of clades [1], supported the previous wealth of historical evidence that YFV was introduced to the American continent from infected mosquitoes and humans boarding the ships at the West African trading posts, commencing ~500 years ago, during the slave trading period. Humans and *Ae. aegypti* vectors were confined together on the ships trading slaves. There are many vivid descriptions of how the slaves were confined below deck under conditions which were ideal for feeding and breeding of the accompanying *Ae. aegypti* and providing the maximum opportunity for YFV to be amplified both in the slaves and the mosquitoes. At the present time, the historical evidence cited above and many phylogenetic observations are coherent with the “Out of Africa” concept: here we summarize the phylogenetic and genetic evidence.

- Five genotypes of YFV have been identified in Africa [138] while two descendant lineages were reported in the Americas [116,145]. As phylogenetic studies showed broadly equivalent rates of nucleotide substitution among African and American isolates [1], the emergence of the five distinct YF genotypes in Africa must have required a longer time span than that of the two American genotypes.
- According to phylogenetic reconstructions, the deepest (i.e., most ancient) phylogenetic node corresponds to the common ancestor of the Angolan and East African lineages, further supporting an African origin for YFV [1].
- The American genotypes are grouped in one phylum that apparently emerged from the West African lineage [51,140,143] and therefore these genotypes are clearly of more recent origin (see Figure 3).
- There is an association between genotypes and the number of imperfectly repeated sequences (RYFs) in the 3′ untranslated region (UTR) of YFV genomes [142,143]. The higher number of RYFs in African YFV sequences also supports the concept of evolution of South American genotypes from West African genotypes notably through the deletion of RYF(s) [142,143,152].
- Phylogenetic reconstructions based on related flaviviral sequences indicate that YFV is most closely related to Old World flaviviruses. Several evolutionary lineages diverged from YFV in Africa (Uganda S, Banzi, Jugra, Wesselsbron) [3,150,153], some of which gave rise to lineages that spread to Asia and Australia (Sepik, Edge Hill viruses) [154]. In contrast, none of these viruses has emerged in America [85].

Other biological lines of evidence point at Africa as the evolutionary cradle of YFV. First, the susceptibility to YFV of South American NHPs. In contrast, YFV is known to be far less pathogenic for African NHPs [2,103,104,150]. Taken together with the historical evidence described earlier this is wholly consistent with the concept that the virus was relatively recently introduced into the New World and evolution towards decreased disease severity has not yet reached the levels seen in Africa. Finally, the African origin of the YFV domestic urban vector, *Ae. aegypti* and the genetic evidence for its introduction to the New World at roughly the same time as the first appearance of YFV in the Americas strongly supports the probability of an “Out of Africa” scenario [119,120,121,150].

## 4. Yellow Fever Virus Epidemiology: A Wide but Not Global, Circulation

YFV is endemic in the tropical regions of sub-Saharan Africa and South-America. Together with Central-America and the Caribbean (where YFV is no longer endemic), these are the regions where the virus has historically caused multiple epidemics. However, YFV outbreaks or epidemics, often involving thousands of humans were also reported in the early 19th century, when trading ships, from the Americas, arrived at ports in the United Kingdom [16] and southern Europe [155].

It was nearly a century before vector-control programs and the extensive use, for decades, of the extremely effective live-attenuated YFV 17D vaccine, were incorporated into large vaccination campaigns [156,157]. Nevertheless, the virus still causes significant outbreaks, as recently reported in Africa and in South America. This is mainly due to the maintenance of the virus through its sylvatic cycle from which it can initiate inter-human transmission cycles by exploiting the forest-to-urban mosquito chain of transmission as defined earlier. Additionally, insufficient vaccine coverage, particularly in regions with limited health infrastructures, indirectly contributed to the continuing outbreaks of yellow fever. Recent studies estimate that between 393 and 472 million people still require vaccination in areas at risk of yellow fever virus transmission, to achieve 80% population coverage (as recommended by World Health Organization) [158]. It is estimated that there are currently around 900 million people at risk in endemic areas, the majority of which live in African countries [156,159]. The epidemiology of yellow fever virus was recently comprehensively reviewed (Monath and Vasconcelos, [149]). We now focus on the most important aspects of the recent epidemics that have taken place since December 2015 in Africa [160,161] and in South America [133,146,162].

### 4.1. In Africa

Most of the annually reported YFV cases occur on the African continent and, more specifically in sub-Saharan Africa, often through large and unpredictable epidemics. Human cases of YF identified in endemic regions of Africa or the Americas invariably arise when infected individuals work in or travel through YF-endemic regions. The epidemiology of YF in Africa involves both bridge and domestic vector species in inter-human transmission cycles (detailed in Section 2).

There is growing concern about the recent resurgence of YFV activity in the tropical regions of Africa where, through human migratory activities, the disease boundaries extend to inhabited rural areas adjacent to urban conurbations. Increasing urbanization results in increasing human and mosquito population densities, thus establishing ideal conditions for YFV emergence and transmission [160]. The 2010 epidemic in Uganda, (the first in 15 years) served as a prelude to the outbreaks that took place in the neighboring countries, Sudan and Ethiopia, in 2013 [86,163]. In December 2015, a large urban epidemic emerged in the Angolan capital Luanda and rapidly spread to the Democratic Republic of Congo, subsequently causing imported cases in Kenya, China, and Mauritania [160,161,164,165]. The outbreaks in Angola and DRC were remarkably large, with more than 7334 suspected cases including 393 deaths reported between December 2015 and December 2016. The widespread dispersion of the epidemic was largely caused by the extensive mobility of people travelling in and out of densely populated capital cities where the urban vector mosquitoes were abundant, thus ensuring efficient dispersion of the virus, to new districts [161]. Moreover, phylogenetic evidence demonstrated an Angolan origin of the circulating virus and that it belonged to the same lineage as that which caused the former Angolan epidemic in 1971. It remains unclear whether the virus circulated through a sylvatic cycle in rural areas, or if it was maintained through silent circulation in the region since the last epidemic in 1988 which ended ~28 years ago [166,167]. Nevertheless, the fact that YFV is still endemic in the region implies that it may re-emerge in the future under favorable conditions [168]. The most recent YFV outbreak in Africa began in Nigeria. After 10 years without any report of YF infection, a total of 112 confirmed cases including 11 deaths have been recorded since September 2017. With more than 10 million people vaccinated by the middle of January 2018 in four out of the seven affected states and as no YF cases have been reported since then, it is not clear if the outbreak is nearing its end [169]. Analyses involving genome sequences of circulating YF strains from this outbreak should lead to new insights into YFV dynamics in this part of West Africa.

### 4.2. In South America

Since the beginning of the 21st century, the PAHO has reported YFV circulation in several South American countries including Brazil, Paraguay, Argentina, Bolivia, Colombia, Venezuela, and Peru [149,165,170].

The largest epizootic of YF registered in Brazil over the last 50 years started in December 2016, in both NHPs and unvaccinated humans inhabiting rural areas of 5 states in South-eastern Brazil (Minas Gerais, São Paulo, Rio de Janeiro, Espírito Santo, and Distrito Federal). In the second half of the 20th century, YFV was endemic in the North (Amazon basin) and Central-West of Brazil. Following a dynamic that started in 1999, the virus is now spreading to the Southern and South-Eastern parts of the country, where it had been absent for decades [114,146,171,172]. Since December 2016, a total of 1380 confirmed cases in NHPs and 2053 humans cases that included 683 deaths (case fatality rate (CFR): 31.7% among confirmed cases) have been reported by the Pan American Health Organization and the Ministry of health [173,174]. Over the past 18 months, case-distribution has followed a seasonal pattern, with most cases being reported during the December 2016–April 2017 and December 2017–March 2018 periods. Importantly, the number of municipalities reporting confirmed YF cases has grown, thereby increasing the population exposed to viral infection from 8.9 to 32 million [175]. The spread of the virus occurs in an area where YFV vaccination is not recommended and may thus be facilitated by low vaccination coverage. Furthermore, this uncommonly large epidemic is associated with YFV variants from the Modern sub-lineage 1E of South America genotype I. Most epidemic strains exhibited several amino acid substitutions within non-structural proteins at locations that are involved in both viral protease and polymerase activities [147,176]. The effect(s) of these changes on the fitness, pathogenicity, and transmissibility of the virus is not yet known. However, both the evidence of positive selection and the coincidence of the emergence of mutations and increased viral dissemination implies a potential positive impact on the spread of the virus [146,147]. Additional phylogenetic and epidemiological studies suggest that the transmission dynamics are primarily sylvatic, with multiple transmission cycles in NHPs, that could inadvertently result in increased human exposure to YFV, indicating a predominant role of NHP-mosquito-human rather than human-mosquito-human transmission during this epidemic [148,177]. Of note, in January, the Evandro Chagas Institute (Brazil) reported the detection of YFV in *Ae. albopictus* from rural areas of Minas Gerais state. This does not imply a significant role of *Ae. albopictus* in YFV transmission in this area but would worth be investigated notably because *Ae. albopictus* mosquitoes from other areas in Brazil (Manaus and Rio states) showed to be susceptible to Brazilian strains of YFV under experimental settings [75]. Yellow fever virus outbreaks involving true urban cycles between humans and *Ae. aegypti* have not been reported recently in South America (see Section 2). However, the presence of dense populations of these mosquitoes in numerous urban centers where most of the inhabitants are non-immune remains a concern in tropical/sub-tropical countries throughout South America [135,136], notably with the emergence of new variants showing increased epidemic potential such as the 1E sublineage of the South American genotype I [145].

### 4.3. In Asia

A feature of YFV that has been discussed for decades is the absence of YFV in Asia in spite of the presence of *Ae. aegypti* mosquito populations, susceptible vertebrate hosts, favorable climatic conditions and substantial exchanges of goods and people with Africa [2,3,51,85,150,178,179]. A wide variety of possible explanations have been proposed, including:

- the relatively low incidence of yellow fever on the east coast of Africa when compared with central and west Africa [76,180]
- the low vector competence of East African and Asian populations of *Ae. aegypti* [126,179,181,182]
- the presence of other flaviviruses, such as dengue virus and viruses in the Japanese encephalitis virus complex which might out-compete YFV or provide an immune background in Asian populations [3,48,51,89,131,179,183,184,185]
- genetic resistance in Asian populations [2]

It seems, from the plethora of factors cited above and, in many publications, that the explanation for the absence of YFV in Asia is likely to be multifactorial. The nature of the interactions between YFV and its primary vector, domestic *Ae. aegypti*, is probably the most important individual determinant of this distinctly different geographic distribution when compared with dengue, Zika, and chikungunya virus all of which share the same primary vector. One thing for sure is that the risk of YFV emerging into Asia is increasing as international trading, urbanization and human mobility, continue to impose a greater impact on arbovirus emergence [85,164,170,186]. One potential saving grace is the fact that yellow fever vaccine is among the safest and most effective of all vaccines—if available to populations concerned.

## 5. Discussion

The ongoing outbreak of yellow fever in humans in Brazil has taken place largely in metropolitan regions of the country, putting around 35.8 million people at risk of YF infection in areas where the occurrence of *Ae. aegypti* maintains an apparently favorable setting for the establishment of urban transmission cycles. Nevertheless, as of 1 April 2018, *Ae. aegypti* still does not appear to be contributing to the current outbreaks. In comparison, following the inadvertent introduction of YFV to China, by infected Chinese workers returning from Angola and DRC, during the 2015–2016 outbreak, the perceived threat of further YFV introductions into *Aedes*-infested regions of Asia has resurfaced. Over the past three years, the global vaccine stockpile has been virtually exhausted and fractional-dosing strategies have now been recommended to increase the number of vaccinated individuals in Brazil and Africa to maximum achievable levels. Considering the speed with which *Ae. aegypti*-transmitted Zika virus and chikungunya virus, dispersed globally, during the past three years, the risk that YFV could become epidemic in densely populated areas of the tropical world other than Latin America and Africa should no longer be ignored. To determine whether such emergence scenarios are pure fantasy or conversely quite possible, it is necessary to fill several gaps that remain in our understanding of the factors that underlie YFV emergence. These may be key in enhancing or restraining the spread of YFV to areas that are, in many respects, apparently suitable for YFV circulation.

In previous publications on YFV, authors have attempted to identify the geographic regions at risk of YFV emergence through modelling based on YF case reporting, the presence of *Ae. aegypti* mosquitoes, and other factors [52,187]. Although these studies are of interest because they provide a global view, one should not ignore that the interaction between the *Ae. aegypti* vector and YFV is complex and varies significantly with the environmental settings. First, as described in this review, *Ae. aegypti* populations are heterogeneous in both genetic and behavioral terms and they may not equally contribute to the circulation of the virus in urban/periurban settings. Second, an important heterogeneity can also be seen among YFV strains in terms of genome and phenotype. The importance of the adequacy between virus and vector has been illustrated by the recent experiments by Couto-Lima and colleagues [75], which have shown that vector susceptibility may vary (i) at the local level, among mosquito populations belonging to the species *Ae. aegypti* and *Ae. albopictus*, recovered from different states in Brazil and (ii), with regard to the strain used for mosquito infection [75]. These results highlight the fact that competence data are only valid for very precise vector-virus interaction pairs. Obviously, large competence surveys involving panels of both mosquitoes and viruses representative of the phenotypic diversity within each group would be laborious, time-consuming, and superfluous in many instances. In contrast, research focusing on specific mosquito-virus associations corresponding to plausible emergence models in areas of the world where the virus is the most likely to emerge are feasible, as proven by the study by Couto-Lima and colleagues [75]. Similar studies, if applied to *Ae. aegypti*-infested regions of Central/North America, Africa, and Southeast Asia would provide more realistic evaluations of the true suitability of these areas for YFV circulation. In the same vein, detailed entomologic surveys coupled to virus isolation/detection and experimental transmission assays would be most valuable to better appraise the effective participation of *Ae. aegypti aegypti* and *Ae. aegypti formosus* subspecies to YFV transmission in nature. More insights into the regional heterogeneity at the level of *Ae. aegypti* populations in both Latin America and Africa might notably enlighten the differences in susceptibility within *Ae. aegypti* populations that have been reported above.

The epidemiology of YFV is difficult to capture in its entirety as it relies essentially on passive surveillance both in human and non-human primates [149]. Improving the detection of YFV infections through serosurveys in humans and non-human primates should ensure greater appreciation of the extent and dynamics of YFV circulation in endemic areas in the future. At the current stage of our understanding, there appear to be small but significant differences between the Old and New World epidemiological characteristics. Epizootics, in South American NHPs, are the primary source of YFV for incidental transmission by sylvatic *Haemagogus*/*Sabethes* mosquitoes to humans. Thus, as the slave trade to the Americas gradually diminished the relatively constant supply of YFV and domestic *Ae. aegypti* to the east coast towns and cities of the Americas effectively dried up, leaving the sylvatic form of YFV as the epizootic reservoir for YFV. Indeed, one could hypothesize that the *Ae. aegypti* eradication campaigns in the New World drove the virus into the forests since when the virus has displayed sylvatic characteristics, only rarely causing epidemics associated with domestic *Ae. aegypti* in urban environments. In other words, in the Americas, during YFV epizootics, the NHPs and their associated sylvatic vectors serve as the direct source for transmission to humans. In contrast, in Africa, YFV epizootics are rarely reported in NHPs. Thus, the link between sylvatic YFV and the urban environment is dependent on an overlapping mosquito-NHP-mosquito-human transmission chain from the forests through the savannah to the rural and peri-urban regions where domestic *Ae. aegypti* becomes the predominant vector. Therefore, in Africa, the connection between NHPs and humans is tenuous. NHP-mosquito-human transmission still serves as a triggering event for epidemics but when urban transmission cycles occur, they do not need to be constantly refueled through spill-overs from NHPs. However, upstream of and during outbreaks, both human and non-human cases of YFV should be watched over with equal attention, as they are equally informative of YFV local occurrence. In addition, a large part of YFV circulation is “silent” as the ratio of inapparent to apparent infection is of approximately 7–12:1 (estimated from field studies [159,188]. Hence, improving our grasp of YFV clinical or sub-clinical cases will increase our understanding of the circulation of YFV in Africa and South America. Overall, strengthening and systematizing YFV case detection should enable improved identification of the areas of viral circulation in most need of vaccination people programs. Currently, 6 million doses of vaccine are maintained annually in the global vaccine stockpile. However, in view of the current fact that YF outbreaks in Africa and Latin America, are constantly expanding [146,149] and given that it takes approximately 12 months to replenish vaccine stocks one is tempted to suggest that the size of the stockpile should be increased significantly. Moreover, in view of the unanticipated global emergence of CHIKV (chikungunya virus) and ZIKV (Zika virus), which are primarily transmitted by the same mosquito vector as YFV, one cannot ignore the possibility that YFV could emerge in Asia at any time. Under such circumstances, it is hard to imagine how the Health Agencies would cope with such a situation.

A possible- and frequently proposed-explanation for the absence of YFV in Asia is the presence of other antigenically-related flaviviruses, including DENV (dengue virus) and members of the JEV (Japanese encephalitis virus) serocomplex. One or more of these viruses could potentially interfere with the spread of YFV through competition in co-infected mosquitoes and/or primates. They might also provide a cross-reactive immune background that reduces infection levels [3,48,51,89,131,179,183,184,185]. Competitive exclusion between YFV and other flaviviruses could be tested experimentally in mosquitoes, and in mammalian hosts (rodents, NHPs). Analyses based on surveillance data could provide additional insights on the significance of co-infection between YFV and other flaviviruses. Investigations on the effect of cross-immunity to other flaviviruses have brought experimental evidence that prior immunization with DENV and/or other flaviviruses allows to reduce viremia upon YFV infection in rhesus macaques and significantly alleviated the symptoms associated to YFV infection in hamsters [184,189]. However, the effect on subsequent mosquito-borne transmission remains to be explored.

Finally, the question of whether there is a genetic resistance of Asian human populations to YFV infection has not been adequately investigated. This is relevant because it has been estimated that around 500,000 people/year travel from China to regions where YFV circulates and thus, are potentially exposed to the YFV, as observed during the 2015–2016 outbreak in Angola [190]. Although it may be quite difficult in many respects, YFV surveillance involving data relative to the origin and/or the genetic background would be most useful to gain insights into this issue.

Answers to at least some of these questions might identify populations most in need of immunization could be valuable in the development of YF vaccination programs in the context of “just-in-time” management of the global vaccine stockpile. The issue of the effectiveness and the strategies for current and future vaccination programs will be discussed in the second section of this review.

## Supplementary Materials

The following are available online at http://www.mdpi.com/2073-4425/9/6/291/s1, Table S1: Yellow fever virus in arthropods.
