# Supplementary material for: What Does the Future Hold for Yellow Fever Virus? (I)
Source: Genes (Basel). 2018 Jun 8;9(6):291. doi: 10.3390/genes9060291 (PMC6027470; doi:10.3390/genes9060291)
Supplement: Supplementary file 1 [file genes-09-00291-s001.pdf]

Table S1. *Yellow fever virus in arthropods*

Region	Potential arthropod vectors of Yellow Fever Virus	Isolation	Laboratory host*	Reference(s)**
Africa	*Aedes (Stegomyia) aegypti* (Linnaeus) *aegypti* (domestic)	Yes	Yes	[1, 2]***
	Aedes (Stegomyia) aegypti (Linnaeus) *formosus* (sylvatic)	Yes	Yes	[1, 2]***
	Aedes (Stegomyia) albopictus (Skuse)	-	Yes	[3, 4]
	Aedes (Stegomyia) africanus (Theobald)	Yes	Yes	[5, 6]
	Aedes (Stegomyia) neoafricanus Cornet, Valade & Dieng	Yes	-	[7, 8]
	Aedes (Stegomyia) opok Corbet & van Someren	Yes	-	[7, 9-12]
	Aedes (Stegomyia) metallicus (Edwards)	Yes	Yes	[13, 14]
	Aedes (Fredwarsius) vittatus (Bigot)	Yes	Yes	[5, 13]
	Aedes (Stegomyia) luteocephalus (Newstead)	Yes	Yes	[7, 8, 15]
	Aedes (Stegomyia) simpsoni (Theobald) *complex (Ae. simpsoni, Ae. lilii, Ae. bromeliae)*	Yes	Yes	[5, 16]
	Aedes (Diceromyia) furcifer-taylori (Edwards) group *(Ae. furcifer, Ae. taylori)*	Yes	Yes	[8, 14]
	Aedes (Aedimorphus) dalzieli (Theobald)	Yes	-	[17]
	Aedes (Aedimorphus) centropunctatus (Theobald)	Yes	-	[17]
	Aedes (Aedimorphus) mcintoshi Huang	Yes	-	[17]
	Aedes (Aedimorphus) stokesi (=*apicoannulatus*) (Edwards)	-	Yes	[15, 18]
	Aedes (Aedimorphus) dentatus (Theobald)	Yes	-	[19]
	Aedes (Aedimorphus) keniensis van Someren	Yes	-	[20]
	Aedes (Aedimorphus) gr tarsalis (Newstead)	Yes	-	[21]
	Anopheles (Cellia) funestus Giles	Yes	-	[17]
	Eretmapodites spp.	Yes	Yes	[8, 15]
	Coquillettidia fuscopennata (Theobald)	Yes	-	[22]
	Mansonia spp.	-	Yes	[23]
	Culex spp.	-	Yes	[24]
	Phlebotomine spp.	Yes	Yes	[13, 25]
	Amblyomma variegatum	Yes	Yes	[26, 27]

Americas	*Aedes (Stegomyia) aegypti* (Linnaeus) *aegypti* (domestic)	Yes	Yes	[1, 2]***
	Aedes (Stegomyia) albopictus (Skuse)	Yes	Yes	[4, 28]
	Haemagogus (*Haemagogus*) *janthinomys* Dyar	Yes	-	[29]
	Haemagogus (*Conopostegus*) *leucocelaenus* (Dyar & Shannon)	Yes	Yes	[30, 31]
	Haemagogus (*Haemagogus*) *spegazzinii* Brethes	Yes	Yes	[32, 33]
	Haemagogus (*Haemagogus*) *albomaculatus* Theobald	Yes	-	[34]
	Haemagogus (*Haemagogus*) *capricornii* Lutz	Yes	Yes	[30, 35]
	Haemagogus (*Haemagogus*) *mesodentatus* Komp & Kumm	Yes	-	[36]
	Haemagogus (*Haemagogus*) *equinus* Theobald	Yes	Yes	[33, 36, 37]
	Haemagogus (*Haemagogus*) *lucifer* (Howard, Dyar & Knab)	Yes	-	[37]
	Haemagogus (*Haemagogus*) *splendens* Williston	-	Yes	[38]
	Sabethes (Sabethoides) chloropterus (Von Humboldt)	Yes	-	[36, 37]
	Sabethes (Sabethes) albiprivus (Theobald)	Yes	Yes	[3, 39]
	Sabethes (Sabethoides) glaucodaemon (Dyar & Shannon)	Yes	-	[40]
	Sabethes (Peytonulus) soperi (Lane & Cerqueira)	Yes	-	[34]
	Sabethes (Sabethes) cyaneus (Fabricius)	Yes	-	[34]
	Aedes (Ochlerotatus) serratus (Felt & Young)	Yes	-	[39]
	Aedes (taeniorhynchus) taeniorhynchus (Wiedemann)	-	Yes	[41]
	Aedes (Ochlerotatus) fulvus (Wiedemann)	Yes	-	[34]
	Aedes (Ochlerotatus) scapularis (Rondani)	Yes	Yes	[39, 41]
	Aedes (Ochlerotatus) fluviatilis (Lutz)	-	Yes	[42]
	Aedes (Ochlerotatus) condolescens Dyar & Knab	Yes	-	[39]
	Anopheles spp.	Yes	-	[37]
	Psorophora spp.	Yes	Yes	[39, 42]
	Mansonia (Mansonia) titillans (Walker)	Yes	-	[39]
	Culex spp.	Yes	Yes	[39, 43]
	Wyeomyia spp.	Yes	-	[39]
	Trichoprosopon frontosus	-	Yes	[44]
	Uranotaenia spp.	Yes	-	[39]

*Arthropod species are reported as lab hosts if they were shown to transmit the virus under experimental conditions. Reported susceptible and non-susceptible species are not specified here.
**References are not comprehensive, when more one than references where available, the earliest one is given.
***The distinction between *Aedes aegypti aegypti* and *Aedes aegypti formosus* is comprehensively described by Brown and colleagues. Reports of isolation/experimental transmission with *Aedes aegypti* species, as reviewed by Germain and colleagues and in Strode 1951, do not specify the subspecies. However, it is commonly accepted that both subspecies are YFV vectors in nature.

References

1. Brown, J.E., et al., *Worldwide patterns of genetic differentiation imply multiple 'domestications' of Aedes aegypti, a major vector of human diseases.* Proc Biol Sci, 2011. **278**(1717): p. 2446-54.
2. Strode, G.K., *Yellow Fever*. Science, ed. N.Y.-L. McGraw-Hill. Vol. 114. 1951. 77-78.
3. Couto-Lima, D., et al., *Potential risk of re-emergence of urban transmission of Yellow Fever virus in Brazil facilitated by competent Aedes populations.* Sci Rep, 2017. **7**(1): p. 4848.
4. Mitchell, C.J., B.R. Miller, and D.J. Gubler, *Vector competence of Aedes albopictus from Houston, Texas, for dengue serotypes 1 to 4, yellow fever and Ross River viruses.* J Am Mosq Control Assoc, 1987. **3**(3): p. 460-5.
5. Philip, C.B., *Preliminary Report of Further Tests with Yellow Fever Transmission by Mosquitoes Other Than Aedes Aegypti.* The American Journal of Tropical Medicine and Hygiene, 1929. **s1-9**(4): p. 267-269.
6. Smithburn, K.C. and A.J. Haddow, *Isolation of yellow fever virus from African mosquitoes.* Am J Trop Med Hyg, 1946. **26**: p. 261-71.
7. Germain, M., et al., *[Sylvatic yellow fever in Africa recent advances and present approach (author's transl)].* Med Trop (Mars), 1981. **41**(1): p. 31-43.
8. Cornet, M., Robin, Y. and Heme, G., *Une poussée épizootique de fièvre jaune selvatique au Sénégal oriental. Isolement du virus de lots de moustiques adultes mâles et femelles.* Med. Malad. Infect., 1979. **9**: p. 63–66.
9. Cordellier, R., *[The epidemiology of yellow fever in Western Africa].* Bull World Health Organ, 1991. **69**(1): p. 73-84.
10. Herve JP, G.M., Geoffroy B. , *Bioécologie comparée d'Aedes (Stegomyia) opok Corbet et Van Someren et A. (S.) africanus (Theobald) dans une galerie forestière du sud de l'Empire Centrafricain II. Cycles saisonniers d'abondance.* . Cah ORSTOM, ser Ent med et Parasitol, 1977. **15**: p. 271-282.
11. Huang, Y.M., *Aedes (Stegomyia) bromeliae (Diptera: Culicidae), the yellow fever virus vector in East Africa.* J Med Entomol, 1986. **23**(2): p. 196-200.
12. Huang, Y.M.S.I., Washington, D.C.), *The subgenus Stegomyia of Aedes in the Afrotropical region. I. The africanus group of species (Diptera: Culicidae).* 1990. **v. 26**.
13. Haddow, A.J., *X.—The Natural History of Yellow Fever in Africa.* Proceedings of the Royal Society of Edinburgh. Section B. Biology, 1969. **70**(3): p. 191-227.
14. Lewis, D.J., T.P. Hughes, and A.F. Mahaffy, *Experimental Transmission of Yellow Fever by Three Common Species of Mosquitoes from the Anglo-Egyptian Sudan.* Annals of Tropical Medicine & Parasitology, 1942. **36**(1-2): p. 34-38.
15. Bauer, *THE TRANSMISSION OF YELLOW FEVER BY MOSQUITOES OTHER THAN AEDES AEGYPTI.* Journal of the American Medical Association 1928. **26**(90): p. 2091-2092.
16. Mahaffy, A.F., et al., *Yellow fever in Western Uganda.* Transactions of The Royal Society of Tropical Medicine and Hygiene, 1942. **36**(1): p. 9-20.
17. Diallo, D., et al., *Patterns of a sylvatic yellow fever virus amplification in southeastern Senegal, 2010.* Am J Trop Med Hyg, 2014. **90**(6): p. 1003-13.
18. Evans, A.M., *Aëdes (Aëdimorphus) Apico-Annulatus Edwards and Yellow Fever: A Correction.* Annals of Tropical Medicine & Parasitology, 1929. **23**(4): p. 521-522.
19. Serie, C., et al., *[Studies on yellow fever in Ethiopia. 5. Isolation of virus strains from arthropod vectors].* Bull World Health Organ, 1968. **38**(6): p. 873-7.
20. Reiter, P., et al., *First recorded outbreak of yellow fever in Kenya, 1992-1993. II. Entomologic investigations.* Am J Trop Med Hyg, 1998. **59**(4): p. 650-6.
21. Ellis, B.R. and A.D. Barrett, *The enigma of yellow fever in East Africa.* Rev Med Virol, 2008. **18**(5): p. 331-46.
22. Kirya, B.G., *A yellow fever epizootic in Zika forest, Uganda, during 1972: Part 1: Virus isolation and sentinel monkeys.* Trans R Soc Trop Med Hyg, 1977. **71**(3): p. 254-60.

23. Philip, C.B., *Studies on Transmission of Experimental Yellow Fevery by Mosquitoes other than Aëdes.* The American Journal of Tropical Medicine and Hygiene, 1930. **s1-10**(1): p. 1-16.
24. Kerr, J.A., *Studies on the Transmission of Experimental Yellow Fever By Culex Thalassius and Mansonia Uniformis.* Annals of Tropical Medicine & Parasitology, 1932. **26**(2): p. 119-127.
25. Smithburn, K.C., A.J. Haddow, and W.H. Lumsden, *An outbreak of sylvan yellow fever in Uganda with Aedes (Stegomyia) africanus Theobald as principal vector and insect host of the virus.* Ann Trop Med Parasitol, 1949. **43**(1): p. 74-89.
26. Cornet, J.P., et al., *[Experimental transmission of the yellow fever virus by the tick Amblyomma variegatum (F.) (author's transl)].* Bull Soc Pathol Exot Filiales, 1982. **75**(2): p. 136-40.
27. Germain, M., et al., *[Isolation of the yellow fever virus from an egg-cluster and the larvae of the tick Amblyomma variegatum].* C R Seances Acad Sci D, 1979. **289**(8): p. 635-7.
28. Saúde, M.d.S.A., *Febre amarela: MS alerta para necessidade de aumentar a vacinação.* 2018.
29. Rawlins, S.C., et al., *Sylvatic yellow fever activity in Trinidad, 1988-1989.* Trans R Soc Trop Med Hyg, 1990. **84**(1): p. 142-3.
30. Shannon, R.C., L. Whitman, and M. Franca, *Yellow Fever Virus in Jungle Mosquitoes.* Science, 1938. **88**(2274): p. 110-1.
31. Bugher, J.C., et al., *The Susceptibility to Yellow Fever of the Vertebrates of Eastern Colombia.* The American Journal of Tropical Medicine and Hygiene, 1941. **s1-21**(2): p. 309-333.
32. Laemmert, H.W.J.F., L. de C.; Taylor, R. M., *An Epidemiological Study of Jungle Yellow Fever in an Endemic Area in Brazil. Part II. Investigations of Vertebrate Hosts and Arthropod Vectors.* Amer. J. Trop. Med., 1946. **26**(No.6): p. 69-79.
33. Waddell, M.B. and R.M. Taylor, *Studies on cyclic passage of yellow fever virus in South American mammals and mosquitoes; marmosets (Callithrix penicillata and Leontocebus chrysomelas) in combination with Aedes aegypti.* Am J Trop Med Hyg, 1946. **26**: p. 455-63.
34. Dégallier N, T.d.R.A., Vasconcelos. PFC, Travassos da Rosa ES, Rodrigues SG, Sá Filho. GC and Travassos da Rosa JFS., *New entomological and virological data on the vectors of sylvatic yellow fever in Brazil.* Brazilian Journal of the Association for Advancement of Science, 1992b. **44**: p. 136-142
35. Waddell, M.B. and H.W. Kumm, *Haemogogus capricornii Lutz as a laboratory vector of yellow fever.* Am J Trop Med Hyg, 1948. **28**(2): p. 247-52.
36. De Rodaniche, E. and P. Galindo, *Isolation of yellow fever virus from Haemagogus mesodentatus, H. equinus and Sabethes chloropterus captured in Guatemala in 1956.* Am J Trop Med Hyg, 1957a. **6**(2): p. 232-7.
37. De Rodaniche, E., P. Galindo, and C.M. Johnson, *Isolation of yellow fever virus from Haemagogus lucifer, H. equinus, H. spegazzinii falco, Sabethes chloropterus and Anopheles neivai captured in Panama in the fall of 1956.* Am J Trop Med Hyg, 1957b. **6**(4): p. 681-5.
38. Anderson, C.R. and E. Osorno-Mesa, *The laboratory transmission of yellow fever virus by Haemagogus splendens.* Am J Trop Med Hyg, 1946. **26**(5): p. 613-8.
39. Vasconcelos, P.F., et al., *Isolations of yellow fever virus from Haemagogus leucocelaenus in Rio Grande do Sul State, Brazil.* Trans R Soc Trop Med Hyg, 2003. **97**(1): p. 60-2.
40. Cardoso Jda, C., et al., *Yellow fever virus in Haemagogus leucocelaenus and Aedes serratus mosquitoes, southern Brazil, 2008.* Emerg Infect Dis, 2010. **16**(12): p. 1918-24.
41. Davis, N.C. and R.C. Shannon, *Studies on Yellow Fever in South America : V. Transmission Experiments with Certain Species of Culex and Aedes.* J Exp Med, 1929. **50**(6): p. 803-8.
42. Whitman, L. and P.C.A. Antunes, *Studies on the Capacity of Various Brazilian Mosquitoes, Representing the Genera Psorophora, Aedes, Mansonia, and Culex, to Transmit Yellow Fever1.* The American Journal of Tropical Medicine and Hygiene, 1937. **s1-17**(6): p. 803-823.
43. Davis, N.C., *Transmission of Yellow Fever Virus by Culex Fatigans Wiedemann. 1.* Annals of the Entomological Society of America, 1933. **26**(3): p. 491-495.
44. Waddell, M.B., *Comparative efficacy of certain South American Aedes and Haemagogus mosquitoes as laboratory vectors of yellow fever.* Am J Trop Med Hyg, 1949. **29**(4): p. 567-75.
